# Supplementary material for: Diurnal Differences in Intracellular Replication Within Splenic Macrophages Correlates With the Outcome of Pneumococcal Infection
Source: Front Immunol. 2022 Jun 2;13:907461. doi: 10.3389/fimmu.2022.907461 (PMC9201068; doi:10.3389/fimmu.2022.907461)
Supplement: Supplementary file 1 [file DataSheet_1.docx]

**
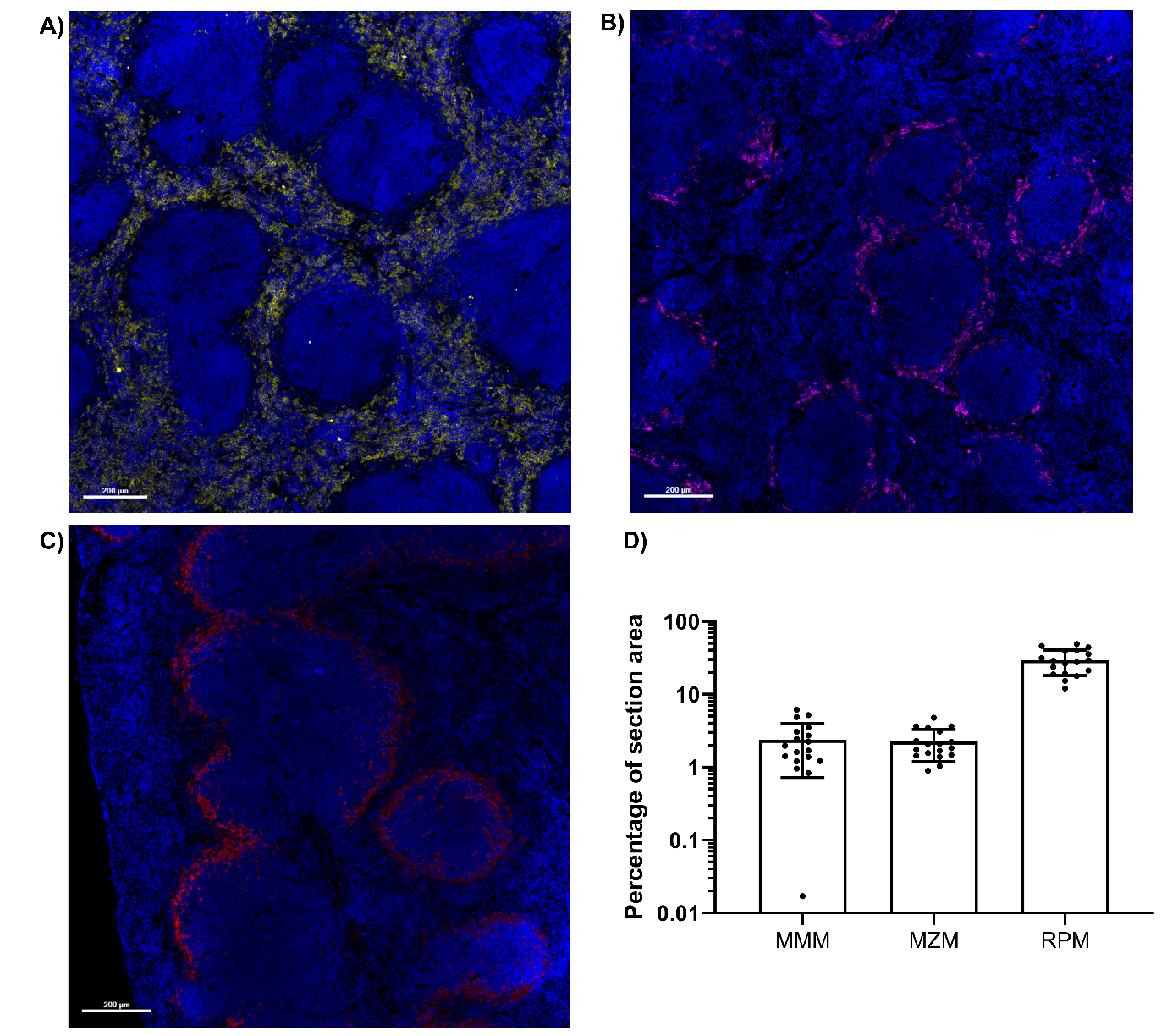
 Supplementary Figure 1. Distribution of macrophage subsets in the murine spleen.** Control rest-phase Balb/cAnN mouse spleens were stained for immunohistochemistry and visualised via scanning confocal microscopy to show **(A)** RPMs (F4/80+; yellow), **(B)** MZMs (MARCO+; magenta) and **(C)** MMMs (CD169+; red). Nuclei are stained using DAPI and appear blue. **D)** Images of Balb/cAnN spleens analysed in Figure 2 (n=54), were analysed to determine the pixel area of MMMs (CD169+), MZMs (MARCO+) and RPMs (F4/80+) as a percentage of the entire section area. Dots represent each analysed spleen. Statistical significance was determined using Dunn’s multiple comparison test (ns; P>0.9999, ****; P<0.0001).

**Supplementary Figure 2. 3D reconstruction animation of Figure 2D.** CD169 is shown in red, pneumococci in green and nuclei in blue. Scale bar represents 5µm.

| **Antibody** | **Specificity** | **Clone** | **Conjugated** | **Catalogue** | **Supplier** |
| --- | --- | --- | --- | --- | --- |
| Purified anti-mouse CD169 (Siglec-1) | MMMs | 3D6.112 | No | 142402 | BioLegend |
| Anti-mouse/human/rat F/80 | RPMs | BM8 | No | 14-4801-82 | Thermo Fisher Scientific |
| Anti-mouse MARCO | MZMs | ED31 | No | MCA1849 | Bio-Rad |
| Anti-type 2 pneumococcal capsule | Type 2 pneumococci | - | No | 16745 | Statents Serum Institut |
| Donkey anti-Rabbit IgG (H+L) | Rabbit IgG (secondary Ab) |  | Alexa Fluor 488 | A-21206 | Thermo Fisher Scientific |
| Goat anti-Rat IgG (H+L) | Rat IgG (secondary Ab) | - | Alexa Fluor 568 | A-11077 | Thermo Fisher Scientific |

**S1 Table. Antibodies used for immunohistochemistry**
